# The Influence of Facial Attractiveness and Advice Feedback on Advisors’ Subsequent Willingness to Continue Giving Advice: Evidence from ERPs

**DOI:** 10.3390/brainsci16080825

**Published:** 2026-08-03

**Authors:** Qian Wang, Shiyu Wan, Meng Liu, Weijun Li

**Affiliations:** 1 Institute of Psychological and Brain Sciences, Liaoning Normal University, Huanghe Road 850, Dalian 116029, China; 2 Key Laboratory of Brain and Cognitive Neuroscience, Liaoning Normal University, Huanghe Road 850, Dalian 116029, China; 3 School of Psychology, Liaoning Normal University, Huanghe Road 850, Dalian 116029, China

**Keywords:** facial attractiveness, advice feedback, FRN, N2

## Abstract

**Highlights:**

**What are the main findings?**

- High-attractiveness faces enhanced P300 and LPP responses across processing.
- Low-attractiveness faces elicited larger FRN and N2 responses to acceptance feedback.

**What are the implications of the main findings?**

- Facial attractiveness and advice feedback jointly shape subsequent advice-giving decisions.
- Attractiveness effects in social decision-making depend on the feedback context.

**Abstract:**

Advice-giving during social interaction is not a one-way flow of information. Instead, it constitutes a dynamic process that includes offering advice, receiving feedback, and adjusting subsequent behaviors. Few studies have examined how advisees’ facial attractiveness and feedback type together shape advisors’ willingness to keep providing advice, as well as the corresponding neural temporal patterns. In this study, we adopted an advice-giving game paradigm. We recorded event-related potentials (ERPs) when 28 participants received feedback and made subsequent advice decisions, to explore the interaction between facial attractiveness and feedback type. Behavioral results showed that both high facial attractiveness and acceptance feedback significantly increased advisors’ willingness to continue giving advice. Moreover, an interaction effect was observed: compared with rejection feedback, the influence of facial attractiveness on willingness to continue advising was relatively weaker under acceptance feedback. ERP results revealed that high facial attractiveness evoked greater P300 and late positive potential (LPP) amplitudes at the feedback reception stage. Under acceptance conditions, low facial attractiveness evoked greater feedback-related negativity (FRN) amplitudes. At the advice decision stage, high facial attractiveness similarly elicited larger P300 and LPP amplitudes, while low facial attractiveness produced greater N2 amplitudes in acceptance trials. These findings indicate that facial attractiveness affects static social evaluation and also shapes the dynamic interaction sequence of giving advice, receiving feedback and forming subsequent decisions. Attractive faces and acceptance feedback may possess greater motivational significance and social salience, thereby promoting advisors’ subsequent willingness to provide advice.

## 1. Introduction

In daily social decision-making, individuals rarely rely solely on their own information to form judgments. Instead, they often seek advice from others, integrate external opinions, and adjust subsequent decisions based on interaction feedback in order to reduce uncertainty and improve judgment quality [1,2,3]. Although advice-giving appears to involve advisors transmitting information to advisees, it actually consists of multiple continuous stages, including advice provision, feedback reception, and subsequent behavioral adjustment, thus reflecting a clear bidirectional interactive nature [1,4]. After providing advice, advisors may receive either acceptance or rejection feedback from advisees, and such feedback can further influence advisors’ evaluations of the interaction partner, as well as their willingness to continue giving advice [5,6,7]. Therefore, advice-giving is better understood as a dynamic interactive process involving “giving advice–receiving feedback–making subsequent decisions.”

Social Exchange Theory (SET) proposes that interpersonal interactions are not merely processes of information exchange but also involve trade-offs between rewards and costs [8]. Individuals adjust their subsequent behaviors accordingly. They rely on recognition, rewards, and relational benefits gained from interactions to make these adjustments. In advice interactions, providing advice may allow advisors to demonstrate competence and gain social recognition, but it also requires investments of time and cognitive resources and may entail psychological costs, such as threats to self-esteem when advice is rejected. Correspondingly, seeking advice is not only an effective strategy for reducing decision uncertainty, but also an important means of social learning through others’ experiences [1]. Thus, advisors’ willingness to continue giving advice is shaped not only by task-related judgments, but also by comprehensive evaluations of feedback outcomes, characteristics of interaction partners, and the value of future relational investment.

Human faces convey abundant social information, and facial attractiveness is among the most rapidly extracted external social cues in interpersonal interaction. A large body of research has shown that attractive individuals are more likely to receive positive trait inferences, social approach tendencies, and advantageous access to resources, a phenomenon commonly referred to as the “beauty premium” or the “what is beautiful is good” effect [9,10,11]. In social decision-making contexts, facial attractiveness influences trust, cooperation, fairness judgments, and resource allocation. For example, in ultimatum and trust games, attractive individuals are more likely to receive favorable evaluations and cooperative opportunities [12,13,14,15]. Moreover, attractive faces themselves can elicit stronger positive emotional and reward-related responses [16,17]. During interactions with attractive advisees, advisors may directly obtain appearance-based rewards from attractive faces. However, previous studies have primarily focused on relatively static social evaluations or one-shot decision-making situations. It remains unclear whether facial attractiveness continues to influence subsequent social investment. This happens after individuals receive acceptance or rejection feedback from interaction partners.

In addition to facial attractiveness, advisees’ acceptance feedback serves as another important factor shaping advisors’ subsequent behaviors. Evidence from advice-giving paradigms suggests that accepted advice is generally processed as positive social feedback and increases advisors’ willingness to provide further suggestions, whereas rejected advice tends to elicit a negative feedback evaluation and greater decisional conflict [7,18]. Moreover, contextual factors such as social hierarchy, interpersonal closeness, and facial expressions further modulate how advisors process feedback outcomes [7,19,20,21]. Taken together, these findings suggest that advisors adjust their subsequent behavioral strategies not only according to whether their advice is accepted, but also by integrating multiple social cues from interaction partners into feedback evaluation and later decision-making processes. Accordingly, the present study aims to address three theoretical questions. First, prior ERP studies have mainly examined feedback processing in isolation. We investigate how advisees’ facial attractiveness, as a positive social cue, is integrated with feedback evaluation during outcome processing. Second, we examine whether attractiveness-linked neural processing extends beyond feedback evaluation to shape the subsequent willingness to continue offering advice. This allows us to test the temporal persistence of social cue processing. Third, we explore how such socially meaningful attributes shape the temporal dynamics of behavioral updating among advisors following feedback reception. This work yields a more dynamic account of the decision-making mechanisms underlying advice-giving interactions.

Event-related potential (ERP) studies provide an important approach for revealing the temporal dynamics of social feedback processing and subsequent decision-making. During the feedback reception stage, feedback-related negativity (FRN), P300, and late positive potential (LPP) are among the most frequently examined ERP components. FRN is a negative wave that typically emerges 200–300 ms after feedback onset. It is mainly linked to reward prediction error, expectancy violation, and the early evaluation of unfavorable outcomes [22,23,24,25]. Prior research shows that FRN responds not only to monetary feedback. It is also sensitive to social evaluation and outcomes of interpersonal acceptance or rejection [7]. The P300 is a positive component associated with attentional resource allocation and salience detection. The LPP is more closely related to sustained processing and emotional processing [26,27]). Stimuli with stronger emotional or reward values usually elicit larger P300 and LPP amplitudes [28,29]. As a social reward cue, facial attractiveness has also been proven to modulate these ERP components [14,15].

In the dynamic interaction sequence of giving advice–receiving feedback–making subsequent decisions, the feedback reception stage primarily reflects advisors’ evaluation and monitoring of advisees’ feedback outcomes. The subsequent decision-making stage mainly reflects the cognitive control and motivational processes. These processes are involved in deciding whether to continue advising interaction partners with different social reward cues. Thus, these two stages involve partially distinct cognitive processes. At the decision-making stage, ERP components associated with conflict monitoring and motivational processing become particularly important. Among them, N2 is commonly regarded as an index of cognitive conflict monitoring and response inhibition, reflecting the degree of competition and uncertainty during behavioral selection [30,31]. P300 and LPP continue to reflect attentional allocation and motivational significance in decision-making contexts [32]. Existing studies suggest that social feedback outcomes can influence individuals’ subsequent behavioral choices and neural responses. For example, acceptance feedback may increase approach tendencies and cooperative willingness, whereas rejection feedback may enhance cognitive conflict and reduce subsequent interpersonal engagement [6,7]. However, relatively few studies have examined how facial attractiveness and social feedback are dynamically integrated during sequential advice-giving interactions. These studies are particularly limited with regard to their influence on subsequent decision-making and its neural mechanisms.

Importantly, social interaction is not solely determined by feedback outcomes. The characteristics of the interaction partner may also continuously influence how individuals interpret social feedback and update subsequent behavior. Attractive faces are generally associated with greater reward value and stronger approach motivation. Therefore, when advisors receive acceptance feedback from attractive advisees, such feedback may possess stronger emotional and motivational significance, thereby enhancing willingness to continue investing in the interaction. In contrast, rejection feedback may weaken or even override the reward advantage associated with attractiveness. Thus, facial attractiveness and feedback type may jointly influence the dynamic process of social decision-making.

The present study employed an advice-giving game paradigm to investigate how facial attractiveness and advice feedback jointly influence advisors’ subsequent decision-making behavior and ERP responses. Specifically, participants first provided advice to advisees with either high or low facial attractiveness, then received either acceptance or rejection feedback, and finally decided whether to continue giving advice. ERP responses were recorded during both the feedback reception stage and the subsequent advice decision stage. Based on previous findings, we proposed the following hypotheses: Behaviorally, both high facial attractiveness and acceptance feedback would increase participants’ willingness to continue giving advice. During the feedback reception stage, high facial attractiveness would elicit larger P300 and LPP amplitudes, reflecting enhanced motivational salience and reward processing; low facial attractiveness, particularly under acceptance conditions, would elicit larger FRN amplitudes, reflecting greater feedback monitoring demands. During the subsequent advice decision stage, high facial attractiveness would elicit larger P300 and LPP amplitudes and smaller N2 amplitudes, indicating reduced decision conflict and enhanced motivational investment. The influence of facial attractiveness would be modulated by feedback type, such that attractiveness effects would be more pronounced under acceptance conditions than under rejection conditions.

## 2. Methods

### 2.1. Participants

Sample size estimation was conducted using G*Power 3.1 [33]. Under the assumptions of a medium effect size (ƒ = 0.25), α = 0.05, and 1 − β = 0.80, at least 24 participants were required for the two-factor repeated-measures ANOVA. A total of 30 college students were recruited for the formal experiment. Two participants were excluded due to excessive EEG artifacts, leaving 28 valid participants (20 females; mean age = 21.36 years, *SD* = 2.04) for the final analyses. All participants were right-handed, had normal or corrected-to-normal vision, and reported no history of neurological or psychiatric disorders. The study was approved by the Research Ethics Committee of Liaoning Normal University. Written informed consent was obtained from all participants prior to the experiment, and appropriate monetary compensation was provided after participation.

### 2.2. Materials

Facial stimuli consisted of neutral-expression face images adopted from a previous study [34] and frontal facial photographs of adults taken by the present research team. A total of 160 facial images were used, including 80 male and 80 female faces. All faces displayed neutral expressions, frontal orientations, and a direct gaze. To minimize the influence of non-facial features on evaluation, all images were standardized using Photoshop CS6 by removing external features such as hair, ears, and beards. In addition, luminance and contrast were normalized using the SHINE toolbox in MATLAB 2024a [35]. All images were finally resized to 260 × 300 pixels.

An independent sample of 20 participants who did not take part in the formal experiment (11 females; Mage = 22.55 years, SD = 1.96) evaluated all face stimuli. Following previous face-perception research [36,37], participants rated each face on attractiveness, trustworthiness, dominance, emotional valence, distinctiveness, and familiarity using 7-point Likert scales, while perceived age was assessed using a continuous numerical estimate. Based on the attractiveness ratings, the stimuli were divided into high- and low-attractiveness groups, each comprising 80 face images, with 40 male and 40 female faces in each group. Paired-sample *t*-tests showed that high-attractiveness faces had significantly higher attractiveness ratings (*M* ± *SD* = 3.60 ± 0.54) than low-attractiveness faces (*M* ± *SD* = 2.63 ± 0.54), *t*(19) = 18.94, *p* < 0.001, indicating a significant difference between the two groups on the attractiveness dimension. High-attractiveness faces had significantly lower perceived age ratings (*M* ± *SD* = 26.16 ± 2.16) than low-attractiveness faces (*M* ± *SD* = 27.47 ± 2.79), *t*(19) = −3.89, *p* < 0.001, indicating a significant difference between the two groups on the perceived age dimension. High-attractiveness faces had significantly higher trustworthiness ratings (*M* ± *SD* = 4.28 ± 0.45) than low-attractiveness faces (*M* ± *SD* = 3.62 ± 0.59), *t*(19) = 7.92, *p* < 0.001, indicating a significant difference between the two groups on the trustworthiness dimension. High-attractiveness faces had significantly higher dominance ratings (*M* ± *SD* = 4.02 ± 0.57) than low-attractiveness faces (*M* ± *SD* = 3.66 ± 0.73), *t*(19) = 2.74, *p* = 0.013, indicating a significant difference between the two groups on the dominance dimension. High-attractiveness faces had significantly higher emotional valence ratings (*M* ± *SD* = 4.29 ± 0.43) than low-attractiveness faces (*M* ± *SD* = 3.56 ± 0.58), *t*(19) = 8.58, *p* < 0.001, indicating a significant difference between the two groups on the emotional valence dimension. No significant difference in distinctiveness ratings was observed between high-attractiveness faces (*M* ± *SD* = 4.14 ± 0.63) and low-attractiveness faces (*M* ± *SD* = 4.01 ± 0.67), *t*(19) = 0.97, *p* = 0.34, indicating no significant difference between the two groups on the distinctiveness dimension. No significant difference in familiarity ratings was observed between high-attractiveness faces (*M* ± *SD* = 3.35 ± 1.05) and low-attractiveness faces (*M* ± *SD* = 3.15 ± 1.15), *t*(19) = 2.03, *p* = 0.06, indicating no significant difference between the two groups on the familiarity dimension. Analyses revealed that the two groups of faces showed no significant differences only in distinctiveness and familiarity, whereas significant differences emerged for all other dimensions. In line with the “what is beautiful is good” effect, attractive faces readily prompt observers to infer positive social traits [9,10,38,39]. Accordingly, we argue that the present facial rating results are theoretically reasonable.

The formal experiment adopted a 2 (facial attractiveness: high vs. low) × 2 (feedback type: acceptance vs. rejection) within-subject design. Within each attractiveness condition, half of the faces were paired with acceptance feedback and the other half with rejection feedback, resulting in four experimental conditions. Each condition contained 40 different faces, with equal numbers of male and female faces. Each face appeared twice during the experiment, yielding 80 trials per condition and 320 trials in total. To avoid carryover effects arising from pairing the same face with different feedback outcomes across trials, each face was consistently paired with a fixed feedback outcome throughout the experiment. This design ensured an equal number of trials and stimuli across conditions while reducing the influence of idiosyncratic characteristics of individual faces.

### 2.3. Experimental Design and Procedure

Before the experiment began, participants were informed that they would complete an advisor–advisee game with another participant located in an adjacent room, in order to enhance the realism of the interactive context. After completing the experiment, participants answered two questions assessing the credibility of the experimental scenario: (1) whether they believed that they had interacted with a real advisee during the task and (2) whether they perceived the entire advisory interaction as genuine and believed that the advisee’s feedback influenced their subsequent decisions [7]. All participants reported believing that the advisee was real and that the feedback influenced their subsequent decision-making. Therefore, no participants were excluded on the basis of the post-experimental credibility check.

During the formal experiment, participants were seated approximately 70 cm from the computer screen. All stimuli were presented using E-Prime 2.0, with a screen refresh rate of 60 Hz. Each trial began with a fixation cross presented for 500 ms, followed by two virtual cards displayed for 1000 ms, and then the monetary values associated with the cards presented for another 1000 ms. Participants were asked to choose the card they believed was more likely to help the advisee obtain a ¥10 reward rather than ¥0 [6], and this choice served as the advice given to the advisee.

**Figure 1:**
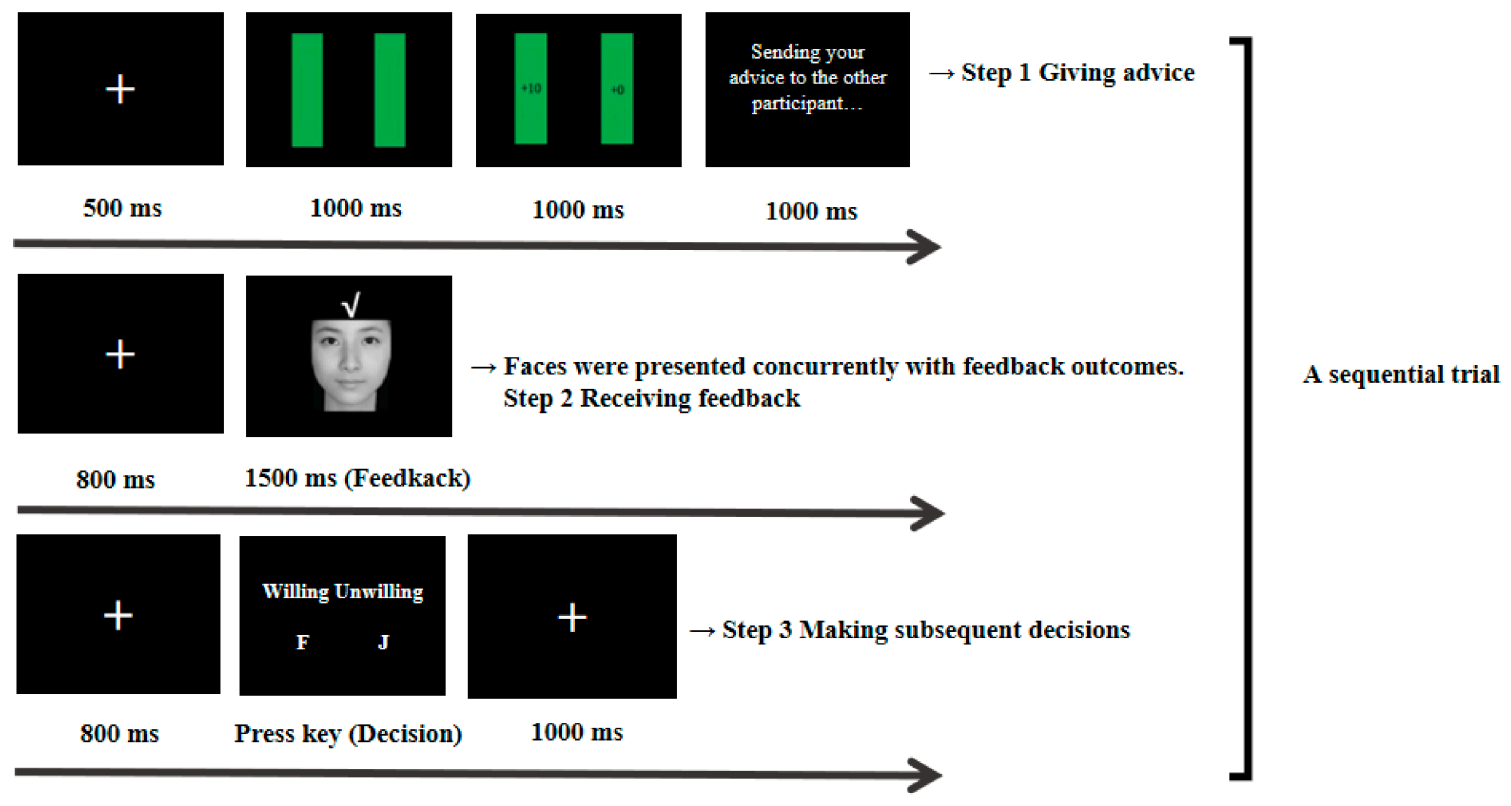
Illustration of one trial in the formal experiment. Step 1 refers to advisors sending advice to the advisees. Step 2 refers to advisors receiving face and feedback information from advisees. Step 3 refers to the advisor’s decision about whether they are willing to continue providing advice to the same advisee. Facial stimuli consisted of neutral-expression face images adopted from a previous study [34], the copyright license certificate has been obtained.

To prevent participants from intentionally selecting disadvantageous cards or making accidental choices, they were told that the computer would automatically send the card with the higher probability of reward to the advisee. The positions of the cards were counterbalanced across the left and right sides. After participants made their choice, the message “Sending your advice to the other participant…” was displayed for 1000 ms to enhance the realism of the interaction context. Next, a fixation cross was presented for 800 ms, followed by the simultaneous presentation of the advisee’s face and feedback outcome for 1500 ms. A “√” indicated that the advisee accepted the advice, whereas a “×” indicated rejection. This phase was defined as the feedback reception stage. After the feedback screen, another fixation cross was presented for 800 ms. Participants then decided whether they were willing to continue providing advice to the same advisee. Participants pressed the “F” key if they were willing to continue and the “J” key if they were unwilling. This phase was defined as the advice decision stage [7]. Finally, a blank screen was presented for 1000 ms to terminate the current trial and reduce carryover effects on the next trial (see Figure 1 for details).

The experiment comprised four blocks of 80 trials each. Within each block, each of the four experimental conditions was presented in 20 trials, with equal numbers of male and female faces in each condition. Trial order was randomized within each block. Before the formal experiment, participants completed 12 practice trials to familiarize themselves with the procedure and to confirm that they understood the task requirements. None of the facial stimuli presented during practice appeared in the formal experiment.

### 2.4. EEG Recording and Data Analysis

EEG data were recorded using a Brain Products actiCHamp system (Brain Products GmbH, Gilching, Germany) with a 64-channel electrode cap arranged according to the extended international 10–20 system. The sampling rate was set at 1000 Hz, and FCz served as the online reference. TP9 and TP10 were placed over the left and right mastoids, respectively. Electrode impedances were maintained below 5 kΩ throughout the experiment. EEG preprocessing was conducted using EEGLAB v.2025.0.40. The continuous EEG data were first downsampled to 500 Hz and filtered using a 0.01 Hz high-pass filter and a 30 Hz low-pass filter. The data were then re-referenced offline to the average of the left and right mastoids. Independent component analysis (ICA) was performed to identify and remove ocular and other stereotypical artifacts. ICA compone0nts were classified using the ICLabel plugin in EEGLAB and were subsequently inspected on the basis of their classifications, scalp topographies, time courses, and spectral characteristics. Components identified as artifacts were removed before epoching. On average, 3.42 artifact-related components were removed per participant. Following ICA correction, epochs containing amplitudes exceeding ±80 μV at any electrode were rejected. The numbers of valid trials retained in each experimental condition are reported below.

For the feedback reception stage, EEG epochs were segmented from 200 ms before to 800 ms after feedback onset, with the 200 ms pre-stimulus interval used as the baseline correction. Based on the prior literature and overall scalp distributions observed in the present data, the FRN was quantified over a frontocentral electrode cluster comprising F1, Fz, F2, FC1, FCz, FC2, C1, Cz, and C2 [22,23,24,25,40]. The P300 and LPP were quantified over a centroparietal electrode cluster comprising C1, Cz, C2, CP1, CPz, CP2, P1, Pz, and P2 [18,32,41]. Repeated-measures ANOVAs with two within-subject factors, facial attractiveness (high vs. low) and feedback type (acceptance vs. rejection), were conducted. To ensure stable ERP waveforms, at least 25–30 valid trials per condition are generally recommended [42]. In the present study, 3.10% of trials were excluded during the feedback reception stage. The average number of valid trials retained per condition was 77.52 (range: 69–80), with no significant differences across conditions (*ps* > 0.05). For the high-attractiveness–acceptance condition, the mean number of retained valid trials was 77.75 (range: 69–80). For the high-attractiveness–rejection condition, the mean number of retained valid trials was 77.39 (range: 69–80). For the low-attractiveness–acceptance condition, the mean number of retained valid trials was 77.54 (range: 69–80). For the low-attractiveness–rejection condition, the mean number of retained valid trials was 77.43 (range: 71–80).

For the advice decision stage, EEG epochs were segmented from 200 ms before to 800 ms after the onset of the decision screen, with the 200 ms pre-stimulus interval serving as the baseline correction. Based on the scalp topographies and previous studies, the ROIs for N2 included F1, Fz, F2, FC1, FCz, FC2, C1, Cz, and C2 [43], whereas the ROIs for P300 and LPP included C1, Cz, C2, CP1, CPz, CP2, P1, Pz, and P2 [18,43]. A total of 2.15% of trials were excluded at this stage. The average number of valid trials retained per condition was 78.28 (range: 58–80), with no significant differences across conditions (*ps* > 0.05). For the high-attractiveness–acceptance condition, the mean number of retained valid trials was 78.36 (range: 69–80). For the high-attractiveness–rejection condition, the mean number of retained valid trials was 77.79 (range: 58–80). For the low-attractiveness–acceptance condition, the mean number of retained valid trials was 78.39 (range: 66–80). For the low-attractiveness–rejection condition, the mean number of retained valid trials was 78.61 (range: 71–80).

The present study further employed the Factorial Mass Univariate Toolbox (FMUT) to conduct cluster-based permutation tests on EEG data [44]. Compared with traditional ERP mean-amplitude analyses, this method simultaneously considers temporal and spatial adjacency relationships among electrodes, thereby improving sensitivity to spatiotemporally continuous effects while controlling for false positives due to multiple comparisons [45,46]. Specifically, exploratory spatiotemporal cluster-based permutation analyses were first conducted across all electrodes and time windows to identify potential significant effects. As a data-driven exploratory procedure, this analysis aims to detect brain regions and time windows with significant spatiotemporal signatures across the whole scalp while rigorously controlling for multiple comparisons. To avoid data-dependent analysis, only significant effects identified in exploratory analyses that satisfied the criteria of predefined regions of interest (ROIs) derived from the prior literature cited above were retained for subsequent analyses. Subsequently, we then carried out further analyses within the ROI and target time windows predefined in the preceding text to examine the specific effects manifested in the corresponding ERP components [47,48]. Adjacent data points meeting the significance threshold of *p* < 0.05 were grouped into the same cluster. Bonferroni correction was applied for simple-effect analyses [45], and the corrected significance threshold for six comparisons was set at *p* < 0.0083. All permutation tests were based on 5000 permutations, and the proportion of permutation statistics exceeding the observed statistic was used as the *p* value.

## 3. Results

### 3.1. Behavioral Results

**Figure 2:**
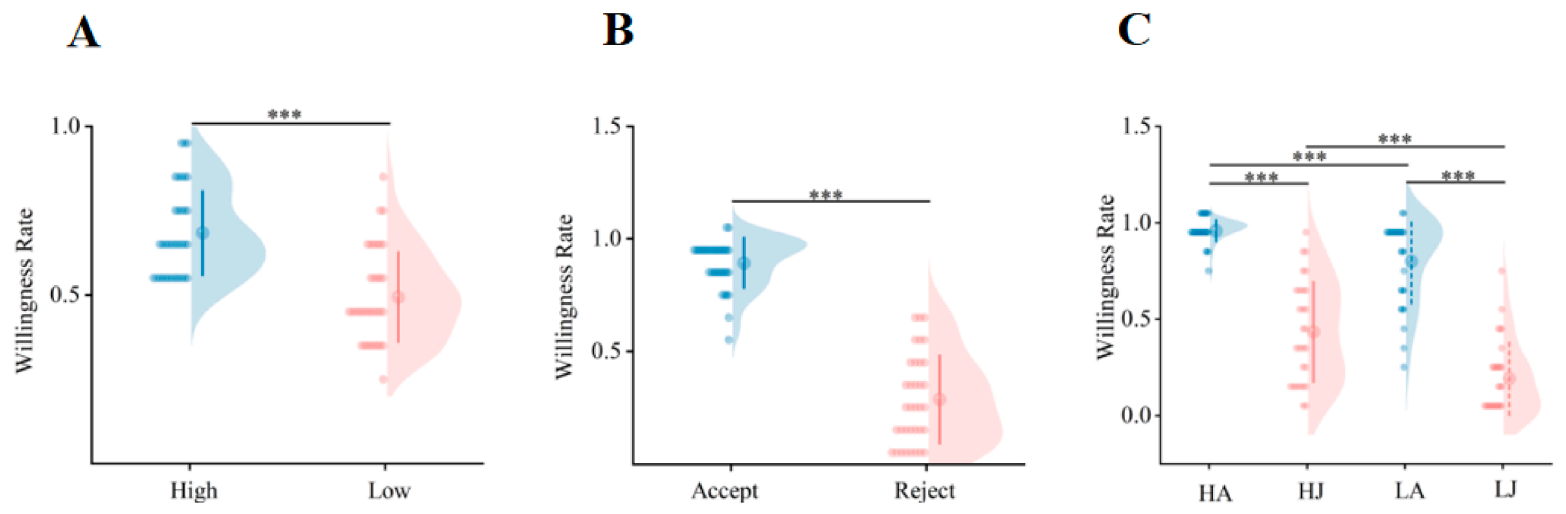
Results for the rate of willingness to continue giving advice. (**A**) Main effect of facial attractiveness. (**B**) Main effect of feedback type. (**C**) Interaction between facial attractiveness and feedback type on willingness rate. HA = high facial attractiveness–acceptance, HJ = high facial attractiveness–rejection, LA = low facial attractiveness–acceptance, LJ = low facial attractiveness–rejection. * *p* < 0.05, ** *p* < 0.01, *** *p* < 0.001.

Behavioral analyses revealed a significant main effect of facial attractiveness, *F*(1, 27) = 34.11, *p* < 0.001, *ηp*^2^ = 0.56. Overall, participants showed a significantly higher willingness to continue giving advice when paired with high-attractiveness advisees (*M* ± *SD* = 0.70 ± 0.13) than with low-attractiveness advisees (*M* ± *SD* = 0.49 ± 0.14). A significant main effect of feedback type was also observed, *F*(1, 27) = 126.38, *p* < 0.001, *ηp*^2^ = 0.82. Regardless of facial attractiveness, participants reported a significantly higher willingness to continue giving advice following acceptance feedback (*M* ± *SD* = 0.88 ± 0.12) than following rejection feedback (*M* ± *SD* = 0.31 ± 0.21). Importantly, the interaction between facial attractiveness and feedback type was significant, *F*(1, 27) = 5.78, *p* = 0.023, *ηp*^2^ = 0.18. Simple-effect analyses showed that, under both high- and low-attractiveness conditions, willingness to continue giving advice was significantly higher following acceptance feedback than rejection feedback, *F*(1, 27) = 101.55, *p* < 0.001, *ηp*^2^ = 0.79; *F*(1, 27) = 123.39, *p* < 0.001, *ηp*^2^ = 0.82. Similarly, under both acceptance and rejection conditions, willingness to continue giving advice was significantly higher for high-attractiveness advisees than for low-attractiveness advisees, *F*(1, 27) = 15.01, *p* < 0.001, *ηp*^2^ = 0.36; *F*(1, 27) = 44.68, *p* < 0.001, *ηp*^2^ = 0.62. Descriptive statistics for each condition were as follows: high-attractiveness–acceptance (0.96 ± 0.06), low-attractiveness–acceptance (0.80 ± 0.22), high-attractiveness–rejection (0.43 ± 0.26), and low-attractiveness–rejection (0.19 ± 0.17) (see Figure 2). Among female participants, willingness rates were 0.95 ± 0.07 in the high-attractiveness–acceptance condition, 0.81 ± 0.19 in the low-attractiveness–acceptance condition, 0.44 ± 0.25 in the high-attractiveness–rejection condition, and 0.19 ± 0.21 in the low-attractiveness–rejection condition. The corresponding rates among male participants were 0.97 ± 0.03, 0.76 ± 0.29, 0.43 ± 0.29, and 0.18 ± 0.14, respectively. Descriptively, male and female participants exhibited the same overall pattern across conditions: willingness was higher following acceptance than rejection feedback and was higher for high-attractiveness than low-attractiveness advisees. However, the similarity of these descriptive patterns alone does not establish the absence of sex-related differences; this would require formal tests involving participant sex.

### 3.2. ERP Results

#### 3.2.1. Feedback Reception Stage

##### Exploratory Analysis Across All Electrodes (0–800 ms)

**Figure 3:**
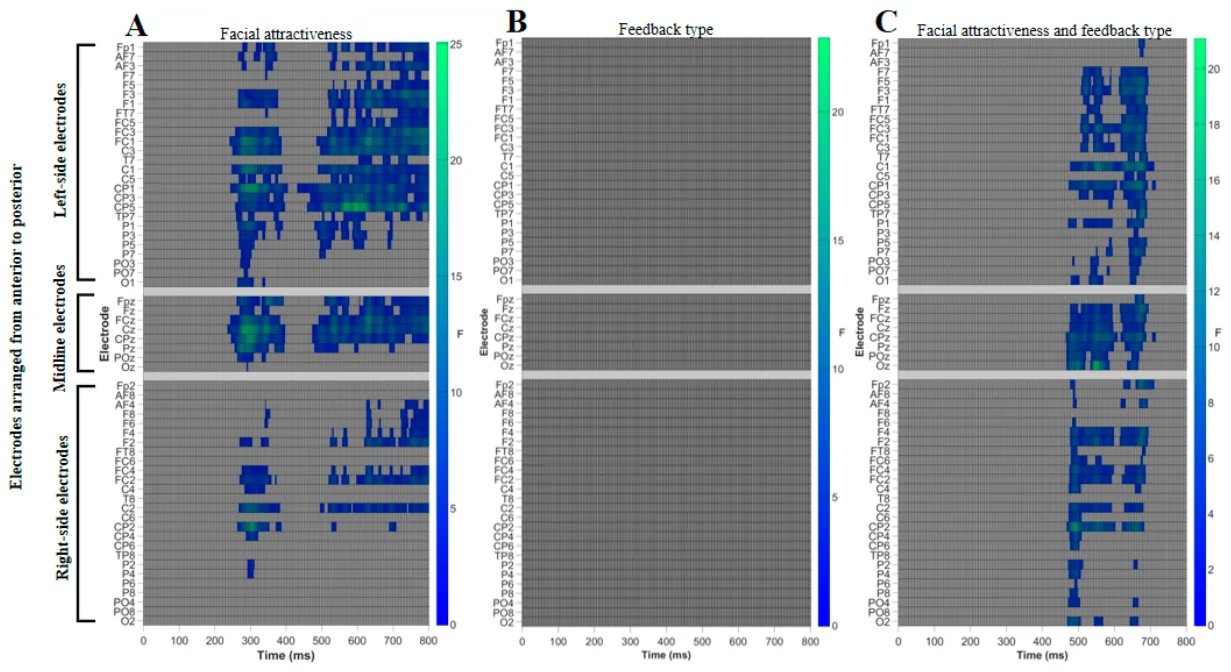
Exploratory analysis of the feedback reception stage. (**A**) Main effect of facial attractiveness on willingness rate. (**B**) Main effect of feedback type on willingness rate. (**C**) Interaction between facial attractiveness and feedback type.

The exploratory analysis revealed significant main effects of facial attractiveness during the feedback reception stage in the 236–404 ms (*p* = 0.0438) and 432–800 ms (*p* = 0.0088) time windows, corresponding to the FRN-, P300-, and LPP-related time ranges. In addition, a significant interaction between facial attractiveness and feedback type emerged in the 466–714 ms time window (*p* = 0.0260), primarily corresponding to the LPP time range (see Table 1 and Figure 3). The brain regions and time windows where significant effects emerged conform to the pre-specified criteria derived from the previous literature. Based on the exploratory results and the previous literature, ROI-based analyses were further conducted within predefined regions of interest and target time windows.

##### ROI-Based Target Time Window Analysis

Based on the valid time windows screened from exploratory analyses for subsequent analyses, together with the ERP waveforms observed at electrodes showing peak F-values, the target time windows for subsequent analyses were defined as 230–270 ms for FRN, 270–340 ms for P300, and 500–800 ms for LPP (see Table 2).

##### FRN (230–270 ms)

Cluster-based spatiotemporal permutation analyses revealed a significant main effect of facial attractiveness, with the peak F-value observed at channel Cz, *F*(1, 27) = 12.29, *p* = 0.0402. Specifically, FRN amplitudes elicited by low-attractiveness faces were significantly larger than those elicited by high-attractiveness faces.

**Figure 4:**
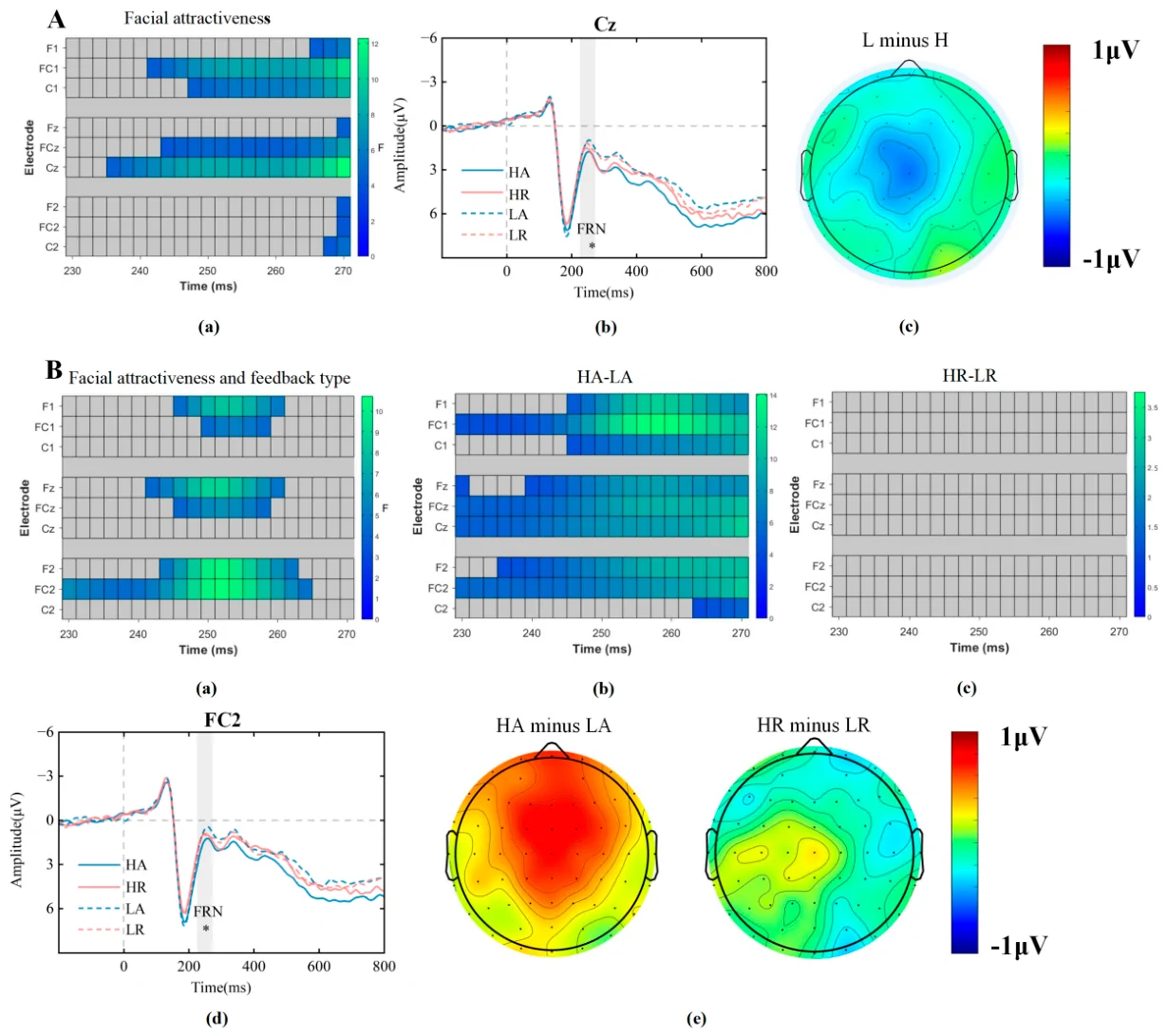
Analysis of the FRN time window in the feedback reception stage. (**A**) Main effect of facial attractiveness: cluster-based permutation test (**a**), waveform at the peak electrode (**b**), and topography (**c**). (**B**) Interaction between facial attractiveness and feedback type: cluster-based permutation test (**a**), simple comparisons (**b**,**c**), waveform at the peak electrode (**d**), and topographies (**e**). * indicates statistically significant differences, and does not represent the level of significance.

A significant interaction between facial attractiveness and feedback type was also observed, with the peak F-value located at channel FC2, *F*(1, 27) = 10.70, *p* = 0.0488. Simple-effect analyses showed that, under the acceptance feedback condition, FRN amplitudes elicited by low-attractiveness faces were significantly larger than those elicited by high-attractiveness faces, *F*(1, 27) = 14.05, *p* = 0.0066. Under the rejection feedback condition, however, no significant difference was found between the two attractiveness conditions, and no significant spatiotemporal clusters emerged. In addition, within both the high- and low-attractiveness conditions, no significant differences were observed between acceptance and rejection feedback (*p* > 0.0610 and *p* > 0.1996, respectively; see Figure 4).

##### P300 (270–340 ms)

**Figure 5:**
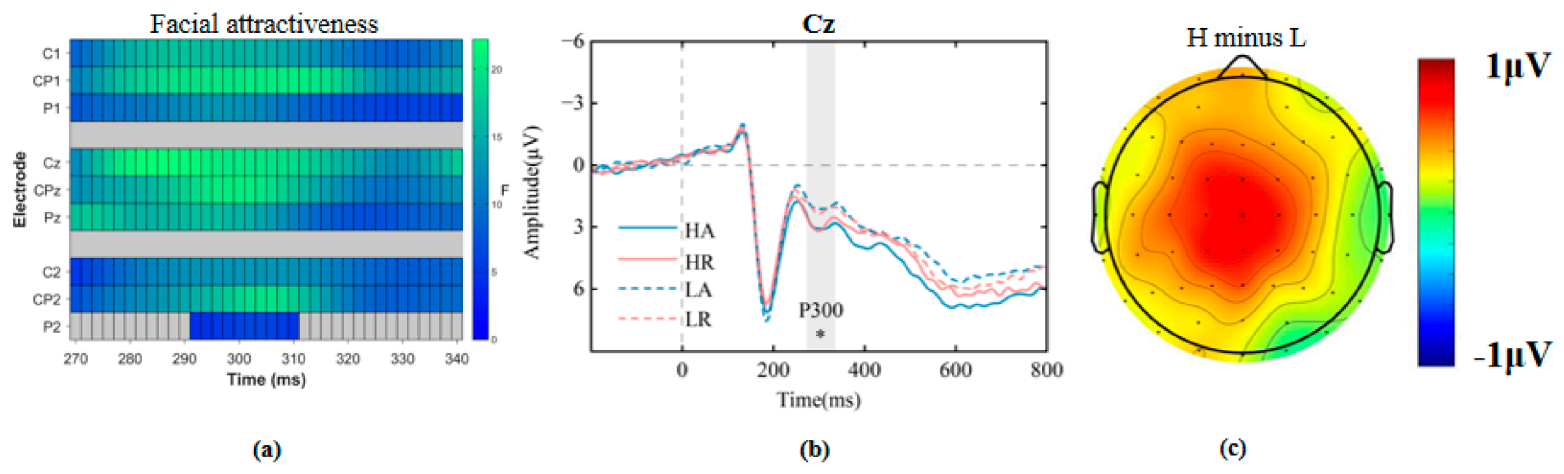
Analysis of the P300 time window in the feedback reception stage. Cluster-based permutation tests for the main effect of facial attractiveness (**a**), waveform at the peak electrode (**b**), and topography (**c**). * indicates statistically significant differences, and does not represent the level of significance.

Cluster-based spatiotemporal permutation analyses revealed a significant main effect of facial attractiveness, with the peak F-value observed at channel Cz, *F*(1, 27) = 22.15, *p* = 0.0002. Specifically, P300 amplitudes elicited under the high-attractiveness condition were significantly larger than those elicited under the low-attractiveness condition (see Figure 5).

##### LPP (500–800 ms)

Cluster-based spatiotemporal permutation analyses revealed a significant main effect of facial attractiveness, with the peak F-value observed at channel Cz, *F*(1, 27) = 16.12, *p* = 0.0030. Specifically, LPP amplitudes elicited under the high-attractiveness condition were significantly larger than those elicited under the low-attractiveness condition. In addition, a significant interaction between facial attractiveness and feedback type was observed, with the peak F-value located at channel C1, *F*(1, 27) = 15.20, *p* = 0.0080.

**Figure 6:**
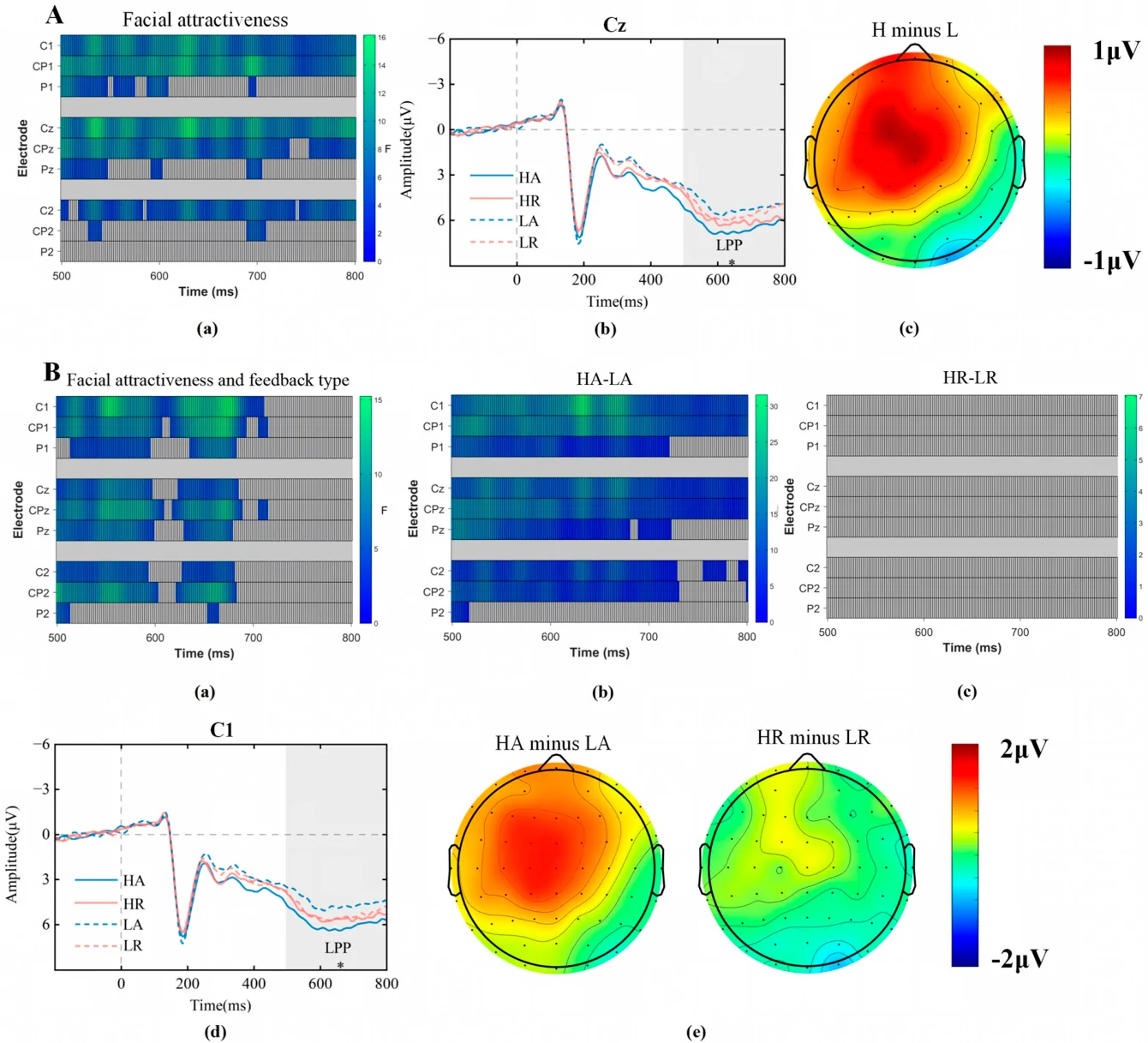
Analysis of the LPP time window in the feedback reception stage. (**A**) Main effect of facial attractiveness: cluster-based permutation test (**a**), waveform at the peak electrode (**b**), and topography (**c**). (**B**) Interaction between facial attractiveness and feedback type: cluster-based permutation test (**a**), simple comparisons (**b**,**c**), waveform at the peak electrode (**d**), and topographies (**e**). * indicates statistically significant differences, and does not represent the level of significance.

Simple-effect analyses further showed that, under the acceptance condition, LPP amplitudes elicited by high-attractiveness faces were significantly larger than those elicited by low-attractiveness faces, *F*(1, 27) = 31.49, *p* = 0.0008. Under the rejection feedback condition, however, no significant difference was observed between the two attractiveness conditions (*p* > 0.4438). Moreover, within both the high- and low-attractiveness conditions, no significant differences were found between acceptance and rejection feedback (*p* > 0.0508 and *p* > 0.1770, respectively; see Figure 6).

#### 3.2.2. Advice Decision Stage

##### Exploratory Analysis Across All Electrodes (0–800 ms)

**Figure 7:**
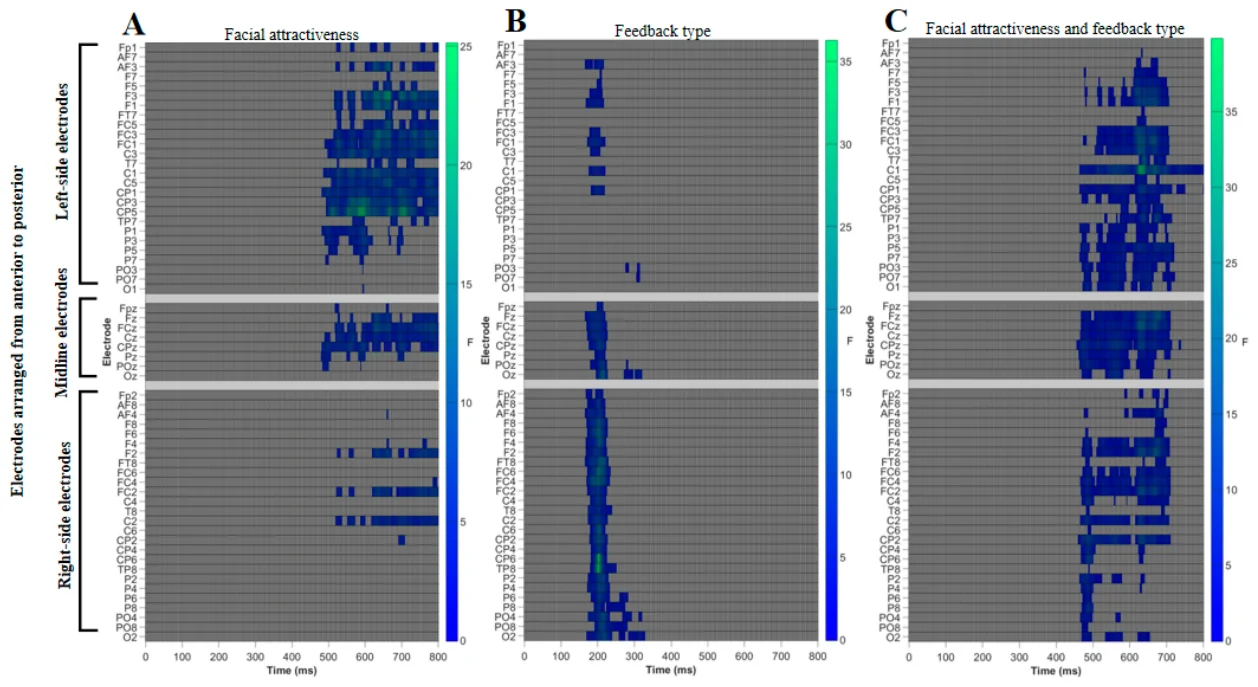
Exploratory analysis of the advice decision stage. (**A**) The main effect of facial attractiveness on willingness rate. (**B**) The main effect of feedback type on willingness rate. (**C**) Interaction between facial attractiveness and feedback type.

Exploratory analyses revealed a significant main effect of facial attractiveness during the advice decision stage within the 480–800 ms time window (*p* = 0.0096), corresponding to the LPP time window. A significant main effect of feedback type was observed within the 166–328 ms window (*p* = 0.0498), corresponding to the FRN time window. In addition, a significant interaction between facial attractiveness and feedback type emerged within the 466–714 ms time window (*p* = 0.0028), which largely overlapped with the LPP time range. The brain regions and time windows where significant effects emerged conform to the pre-specified criteria derived from the previous literature. Based on the exploratory findings and the previous literature, ROI-based analyses were subsequently conducted within predefined regions of interest (ROIs) and target time windows (see Table 3 and Figure 7).

##### ROI-Based Target Time Window Analysis

Based on the valid time windows screened from exploratory analyses for subsequent analyses, together with the previous literature and the observed ERP waveforms at electrodes showing peak F-values, the target time windows for subsequent analyses were defined as 230–270 ms for N2, 270–340 ms for P300, and 500–800 ms for LPP (see Table 4).

##### N2 (230–270 ms)

**Figure 8:**
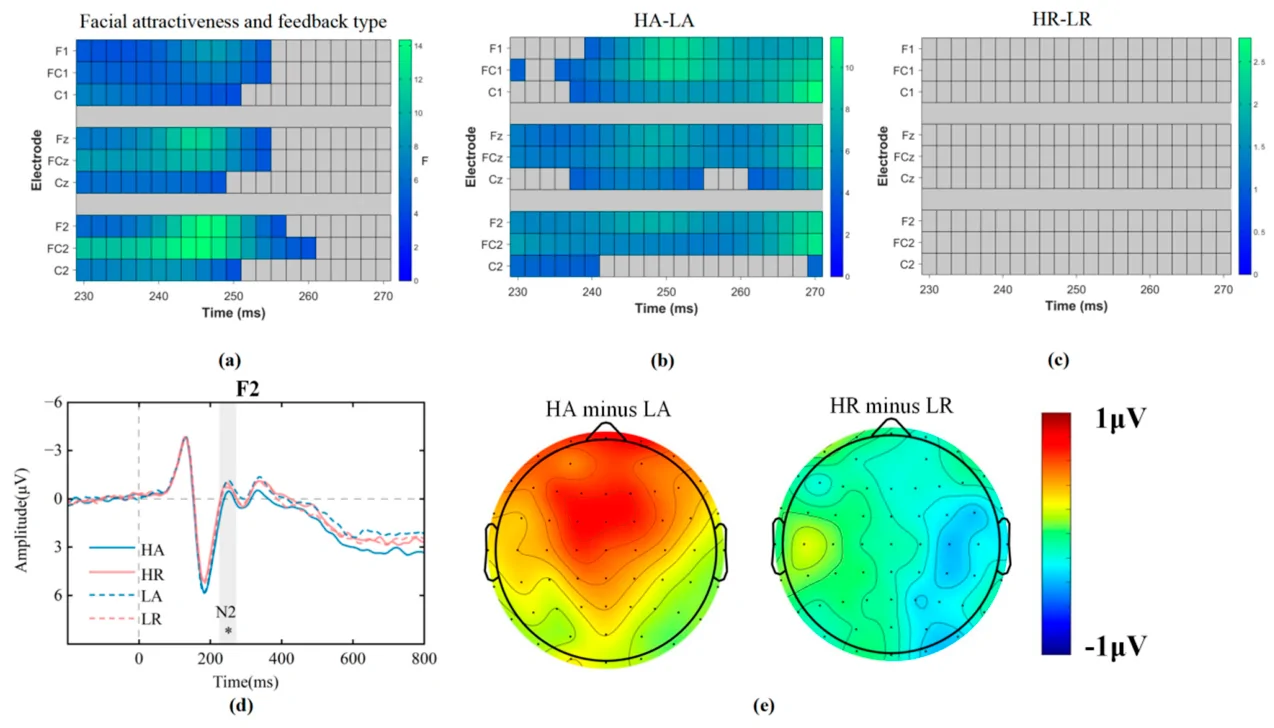
Analysis of the N2 time window in the advice decision stage. Interaction between facial attractiveness and feedback type: cluster-based permutation test (**a**), simple comparisons (**b**,**c**), waveform at the peak electrode (**d**), and topographies (**e**). * indicates statistically significant differences, and does not represent the level of significance.

Cluster-based spatiotemporal permutation analyses revealed a significant interaction between facial attractiveness and feedback type, with the peak F-value observed at channel F2, *F*(1, 27) = 14.32, *p* = 0.0056. Simple-effect analyses showed that, under the acceptance condition, N2 amplitudes elicited by low-attractiveness faces were significantly larger than those elicited by high-attractiveness faces, *F*(1, 27) = 11.41, *p* = 0.0070. Under the rejection condition, however, no significant difference was found between the two attractiveness conditions, and no significant spatiotemporal clusters emerged. In addition, within both the high- and low-attractiveness conditions, no significant differences were observed between acceptance and rejection feedback (*p* > 0.0604 and *p* > 0.3112, respectively; see Figure 8).

##### P300 (270–340 ms)

**Figure 9:**
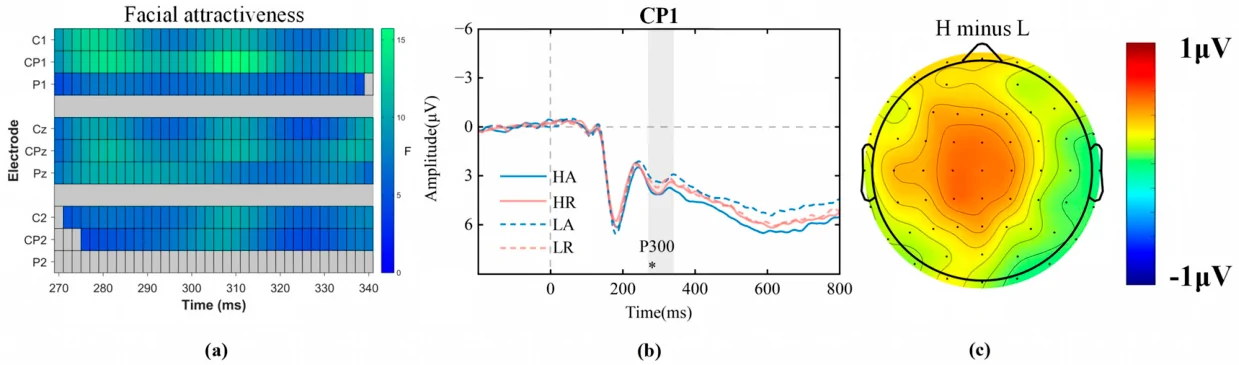
Analysis of the P300 time window in the advice decision stage. Cluster-based permutation test for the main effect of facial attractiveness (**a**), waveform at the peak electrode (**b**), and topography (**c**). * indicates statistically significant differences, and does not represent the level of significance.

Cluster-based spatiotemporal permutation analyses revealed a significant main effect of facial attractiveness, with the peak F-value observed at channel CP1, *F*(1, 27) = 15.68, *p* = 0.0016. Specifically, P300 amplitudes elicited under the high-attractiveness condition were significantly larger than those elicited under the low-attractiveness condition (see Figure 9).

##### LPP (500–800 ms)

**Figure 10:**
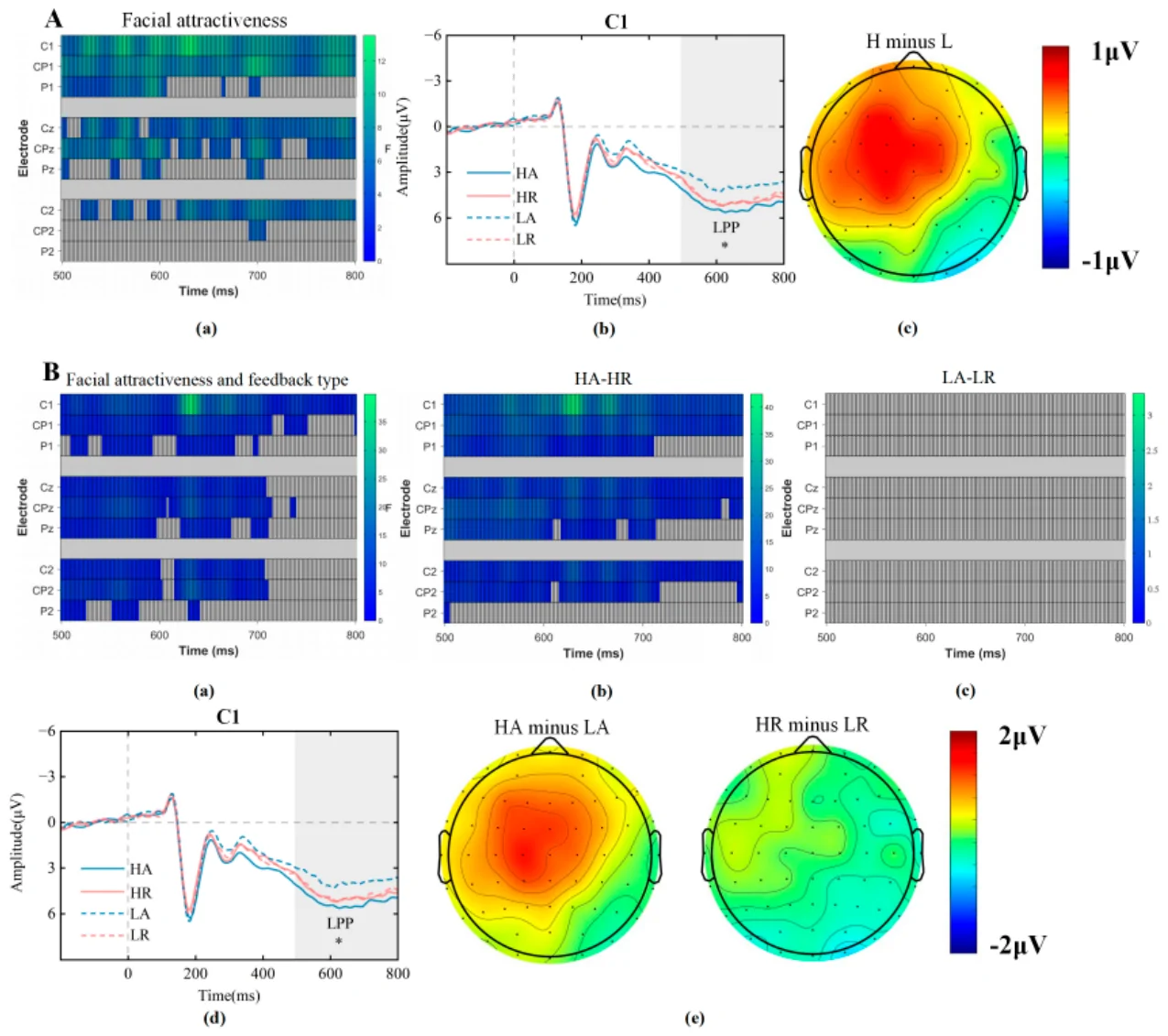
Analysis of the LPP time window in the advice decision stage. (**A**) Main effect of facial attractiveness: cluster-based permutation test (**a**), waveform at the peak electrode (**b**), and topography (**c**). (**B**) Interaction between facial attractiveness and feedback type: cluster-based permutation test (**a**), simple comparisons (**b**,**c**), waveform at the peak electrode (**d**), and topographies (**e**). * indicates statistically significant differences, and does not represent the level of significance.

Cluster-based spatiotemporal permutation analyses revealed a significant main effect of facial attractiveness, with the peak F-value observed at channel C1, *F*(1, 27) = 13.49, *p* = 0.0034. Specifically, LPP amplitudes elicited under the high-attractiveness condition were significantly larger than those elicited under the low-attractiveness condition. In addition, a significant interaction between facial attractiveness and feedback type was observed, with the peak F-value also located at channel C1, *F*(1, 27) = 39.77, *p* = 0.0022. Simple-effect analyses further showed that, under the acceptance condition, LPP amplitudes elicited by high-attractiveness faces were significantly larger than those elicited by low-attractiveness faces, *F*(1, 27) = 42.36, *p* = 0.0004. Under the rejection condition, however, no significant difference was observed between the two attractiveness conditions, and no significant spatiotemporal clusters emerged. Moreover, within both the high- and low-attractiveness conditions, no significant differences were found between acceptance and rejection feedback (*p* > 0.0990 and *p* > 0.0176, respectively; see Figure 10).

## 4. Discussion

Using an advice-giving game paradigm, the present study investigated how facial attractiveness and advice feedback jointly influence advisors’ subsequent decision-making at both the behavioral and ERP levels. Unlike previous studies that mainly focused on static social evaluation or one-shot social decisions, the current study embedded facial attractiveness into a dynamic interaction chain involving “giving advice–receiving feedback–making subsequent decisions.” The findings suggest that advisors’ subsequent advice-giving behavior is not determined solely by feedback outcomes, but is jointly shaped by feedback valence and the facial attractiveness of the interaction partner. Overall, high facial attractiveness functioned as a positive social reward cue, whereas acceptance feedback served as a signal of interpersonal approval. Together, these factors influenced advisors’ willingness to continue investing in the interaction and modulated the corresponding neural processes.

At the behavioral level, acceptance feedback significantly increased participants’ willingness to continue giving advice, consistent with previous advice-interaction studies [7]. Acceptance of one’s advice implies that the interaction partner acknowledges the advisor’s judgment, thereby providing positive social feedback and reinforcing self-efficacy [49]. Such feedback may also increase intrinsic satisfaction and perceived relational benefits derived from advice-giving. In contrast, rejection feedback may be processed as a signal of social disapproval, leading advisors to perceive that their cognitive and self-esteem investments were not rewarded, thereby reducing subsequent willingness to continue advising. In addition, attractive faces also increased willingness to continue giving advice. This finding suggests that advisors integrate not only feedback outcomes but also external social cues and their associated reward value. They do so when deciding whether to maintain social investment [9,10,50]. Our initial hypothesis posited that acceptance feedback and high facial attractiveness would jointly facilitate participants’ sustained advice-giving behavior, rendering the positive effect of facial attractiveness more salient following acceptance feedback. However, the behavioral analyses revealed a significant interaction between facial attractiveness and feedback type that did not support this directional hypothesis. Specifically, the influence of facial attractiveness was attenuated under acceptance feedback but became more pronounced following rejection feedback. Thus, the behavioral findings suggest that facial attractiveness exerted a stronger influence on subsequent advice decisions after rejection than after acceptance.

During the feedback reception stage, low-attractiveness faces elicited larger FRN amplitudes. Previous research has associated the FRN with early feedback evaluation, expectancy violation, and outcome monitoring [22,23,24,25]. Accordingly, the present findings suggest that facial attractiveness modulates early neural responses during feedback evaluation. Further analyses indicated that this modulation was specific to the acceptance condition: low-attractiveness faces elicited significantly larger FRN amplitudes than high-attractiveness faces following acceptance feedback, whereas no significant attractiveness effect was observed following rejection feedback. These findings indicate that early feedback-related neural activity was influenced not only by feedback valence but also by the social characteristics of the interaction partner. One possible interpretation is that acceptance feedback delivered by attractive advisees may have been perceived as more congruent with participants’ prior social expectations or more socially positive, thereby reducing the magnitude of early evaluative processing. In contrast, acceptance feedback from less attractive advisees may have required greater early evaluative processing, resulting in relatively larger FRN amplitudes. However, because the present study did not directly assess participants’ expectations, perceived reward value, or other social evaluations associated with facial attractiveness, these interpretations should be regarded as plausible explanations rather than definitive accounts of the observed ERP effects. No significant modulatory effect of facial attractiveness was observed under rejection feedback. One possible explanation is that rejection feedback constitutes a highly salient negative social signal that may dominate early feedback evaluation, thereby reducing the influence of facial attractiveness during this processing stage [7,17]. Nevertheless, the present data do not permit us to distinguish this interpretation from alternative explanations, and future studies incorporating direct measures of expectancy, perceived reward value, or related social evaluations will be necessary to clarify the mechanisms underlying these effects.

P300 is commonly associated with attentional allocation, emotional evaluation, and reward processing [18,26,27,51]. The present findings regarding P300 and LPP further reveal that attractive faces elicit enhanced mid–late positive ERP components during feedback reception. This suggests that such faces may capture greater attentional resources and possess higher motivational salience and reward value. In particular, during the LPP time window, attractive faces elicited significantly larger LPP amplitudes than low-attractiveness faces under acceptance feedback, whereas no significant difference was found under rejection feedback. LPP is generally considered to reflect sustained attentional allocation and elaborative processing toward emotionally and motivationally salient stimuli and is particularly sensitive to highly arousing, rewarding, and socially meaningful information [28,29,52,53]. Previous studies have shown that attractive faces are more likely to elicit positive trait inferences and social approach tendencies, known as the “what is beautiful is good” effect [9,10]. Attractive faces also possess stronger reward and emotional arousal value, thereby attracting greater attentional resources and eliciting enhanced late positive components [14,16,52,53]. Thus, when attractive advisees accepted the advice, advisors not only received positive feedback, indicating that their advice had been adopted, but also received approval from a socially rewarding interaction partner. The consistency between feedback outcome and partner reward value likely enhanced motivational attention and sustained evaluative processing, resulting in larger LPP amplitudes. In contrast, when the feedback was rejection, the negative meaning of “advice not being accepted” may have weakened or even overridden the reward advantage associated with facial attractiveness, shifting processing focus toward the rejection outcome itself. Therefore, the influence of facial attractiveness on LPP was not unconditional but depended on feedback valence. Under positive feedback conditions, high facial attractiveness amplified perceived social reward and relational investment value. However, under negative feedback conditions, the salience of rejection reduced the impact of attractiveness cues on late-stage processing. The present study aimed to investigate how advice feedback and facial attractiveness jointly influence interpersonal interactions. Accordingly, our interpretations of the P300 and LPP findings were guided by the observed ERP patterns and the previous literature. However, because these ERP components are not process-specific neural markers, they may also be influenced by attentional allocation, expectancy, stimulus novelty, task relevance, and other cognitive factors. Therefore, the proposed interpretations should be regarded as plausible explanations rather than definitive accounts of the underlying psychological mechanisms.

At the advice decision stage, low-attractiveness faces elicited significantly larger N2 amplitudes than high-attractiveness faces under acceptance conditions, whereas no significant difference was observed under rejection conditions. N2 is typically associated with conflict monitoring, response inhibition, and cognitive control, particularly reflecting the monitoring of competing responses or decision uncertainty during behavioral selection [7,30,31,43,54]. In the present study, acceptance feedback itself increased advisors’ tendency to continue giving advice; however, this willingness was still modulated by facial attractiveness. For attractive advisees, acceptance feedback and facial attractiveness may have constituted congruent, positive social cues. Participants exhibited smaller N2 amplitudes under this condition. One possible explanation is that this pattern reflects reduced conflict control demands in subsequent decision-making processes. In comparison, trials with feedback paired with low-attractiveness faces elicited larger N2 amplitudes, indicating that this condition requires more cognitive control resources. However, because the present study did not directly assess participants’ motivational state, perceived value of future interaction, or conflict-monitoring processes, these interpretations should be regarded as plausible explanations that are consistent with, but not uniquely supported by, the present data. Alternative cognitive mechanisms may also account for the observed N2 modulation. Under rejection conditions, no significant attractiveness-related differences emerged. This may occur because rejection feedback had already reduced the motivation to continue advising. This may enable feedback valence to dominate neural processing underlying subsequent behavioral selection, while attenuating the impact of facial attractiveness on conflict-monitoring and cognitive control processes.

In addition, attractive faces elicited larger P300 and LPP amplitudes during the advice decision stage, indicating that facial attractiveness continued to modulate later neural processing during subsequent behavioral choices. One possible interpretation is that attractive faces received greater attentional allocation or motivational salience; however, these mechanisms were not directly assessed and cannot be uniquely inferred from the present ERP findings. Although the feedback reception stage and the advice decision stage both belonged to the same dynamic interaction chain, they involved different processing emphases. The feedback reception stage primarily reflected immediate evaluation and monitoring of social feedback outcomes, focusing on whether the advice was accepted and whether the outcome matched expectations. In contrast, the advice decision stage involved deciding whether to continue investing in the relationship based on prior feedback. It shifts the focus from outcome evaluation to future relational value assessment, behavioral control, and conflict monitoring [55,56,57]. Despite these differences, facial attractiveness exerted a continuous modulatory influence across both stages, consistently enhancing P300 and LPP amplitudes. This suggests that attractiveness cues not only influence immediate social feedback processing but also extend into subsequent behavioral updating and relational investment decisions. Interpretations regarding ERP components at this stage are also reasonable speculations derived from the experimental objectives of the present study.

Taken together, the behavioral and ERP findings suggest that facial attractiveness and feedback outcomes jointly influenced advice-related processing, although the specific interaction patterns differed across measures. Feedback exerted a stronger influence on final advice decisions, whereas facial attractiveness modulated neural processing during feedback evaluation and subsequent decision-making.

Several limitations should be acknowledged. First, the sample size was relatively small, and female participants constituted a large proportion of the sample. Although behavioral data indicated that the findings from participants of different sexes were comparable, potential influences arising from sex cannot be fully ruled out. Future studies should recruit larger and more gender-balanced samples to improve the stability and generalizability of the findings. Second, although the laboratory-based interaction paradigm enhanced ecological validity through task instructions and feedback settings, it still cannot fully replicate real interpersonal interactions. Future studies could incorporate real dyadic interactions, trial-level behavioral modeling, and subjective emotional ratings to more precisely reveal the relationships among facial attractiveness, feedback outcomes, and subsequent social investment. Third, face stimuli were repeatedly presented in this study. Repeated exposure to faces may elicit familiarity effects in participants and thereby reduce statistical power. In addition, faces with different attractiveness levels differed significantly on dimensions reflecting positive personal traits such as trustworthiness. Although attractive faces are generally perceived to possess more positive attributes, we cannot fully rule out the interference caused by such dimensional differences on the experimental results. Fourth, personality traits exhibit complex interactive relationships and cannot be fully controlled in experimental settings. Since individual personality differences were not directly measured in the present study, such unaccounted variations may serve as a potential source of confounding effects in the current results. Finally, no stable effect of feedback type was observed on P300, and the interpretation of this finding remains exploratory. Future studies could further manipulate feedback intensity, feedback credibility, and relationship context to clarify these effects.

## 5. Conclusions

Using an advice-giving paradigm, the present study demonstrated that facial attractiveness and advice feedback jointly influence advisors’ subsequent decision-making. Behavioral results showed that both high facial attractiveness and acceptance feedback increased willingness to continue giving advice. ERP findings further revealed that, during the feedback reception stage, high facial attractiveness enhanced mid-to-late attentional and motivational processing indexed by P300 and LPP. Low facial attractiveness elicited stronger FRN amplitudes under acceptance conditions. During the advice decision stage, high facial attractiveness similarly enhanced P300 and LPP amplitudes and reduced N2-related cognitive conflict under acceptance conditions. Overall, the what-is-beautiful-is-good stereotype and feedback modulation operate not merely within static evaluative settings, but also dynamically influence feedback processing and subsequent behavioral decisions in social interaction.

## Figures and Tables

**Table 1:** Summary of the main effect of facial attractiveness and the interaction effect between facial attractiveness and feedback type (*p* < 0.05) during the feedback reception stage of the exploratory analyses.

Test	Timing	Electrodes	Peak
Main effect of facial attractiveness Interaction effect between facial attractiveness and feedback type	236–404 ms 432–800 ms 466–714 ms	Cz, FC1, CP1, FCz, CPz, C1, C3, Pz, CP5, FC3, TP7, Fpz, O1, CP2, P1, POz, F3, F1, CP3, AF7, P3, C2, C5, P5, Fz, FC2, F2, PO3, P7, AF3, FC4, Fp1, PO7, C4, CP4, Oz, P4, P2, F6, F7, F8, AF4, FT7 CP1, CP5, CP3, Cz, CPz, Pz, P1, FC1, P3, C1, FCz, P7, C3, C2, P5, TP7, Fpz, FC3, Fp1, F3, FC2, F1, Fz, F2, AF3, C5, CP2, FC5, FC4, FT7, F4, AF7, F5, T7, F7, F8, AF4, F6 CPz, Pz, CP2, POz, Oz, O2, P1, P2, PO4, C4, P4, CP1, C2, C6, P8, Cz, FC2, FC4, CP4, Fp2, F4, Fz, CP6, FCz, F2, C1, O1, FC6, FT8, PO3, F6, AF4, AF8, P6, CP3, C3, FC3, F3, FC5, F5, FC1, F7, F1, FT7, PO7, P7, CP5, P5, C5, T7, TP7, P3, Fpz, Fp1, AF7	CZ at 286 ms (*F* = 22.15 *p* = 0.0438) CP5 at 596 ms (*F* = 25.02 *p* = 0.0088) OZ at 548 ms (*F* = 21.05 *p* = 0.0260)

**Table 2:** Summary table of the main effects, interactions (*p* < 0.05) and follow-up comparisons (*p* < 0.0083) for the feedback reception stage.

Test	Timing	Electrodes	Peak
Facial attractiveness main effect (FRN) Facial attractiveness main effect (P300) Facial attractiveness main effect (LPP) Facial attractiveness and feedback type interaction effect (FRN) Acceptance: High facial attractiveness compared to low facial attractiveness Reject: High facial attractiveness compared to low facial attractiveness Facial attractiveness and feedback type interaction effect (LPP) Acceptance: High facial attractiveness compared to low facial attractiveness Reject: High facial attractiveness compared to low facial attractiveness	236–270 ms 270–340 ms 500–800 ms 230–264 ms 500–714 ms	Cz, FC1, FCz, C1, F1, C2, Fz, FC2, F2 Cz, CPz, Pz, CP1, CP2, P1, C1, C2, P2 Cz, CPz, Pz, CP1, CP2, P2, C1, C2, P1 FC2, Fz, F2, FCz, F1, FC1 Cz, CPz, Pz, CP1, CP2, P2, C1, C2, P1	CZ at 270 ms (*F* = 12.29 *p* = 0.0402) CZ at 286 ms (*F* = 22.15 *p* = 0.0002) CZ at 630 ms (*F* = 16.12 *p* = 0.0030) FC2 at 252 ms (*F* = 10.70 *p* = 0.0488) FC2 at 252 ms (*F* = 14.05 *p* = 0.0066) N/A C1 at 674 ms (*F* = 15.20 *p* = 0.0080) C1 at 674 ms (*F* = 31.49 *p* = 0.0008) N/A

**Table 3:** Summary of the facial attractiveness main effect, feedback type main effect and the interaction effect between facial attractiveness and feedback type (<0.05) for the advice decision stage of the exploratory analyses.

Test	Timing	Electrodes	Peak
Facial attractiveness main effect Feedback type Facial attractiveness and feedback type interaction effect	480–800 ms 166–328 ms 466–714 ms	Pz, CP1, P1, CPz, POz, C1, P3, Cz, FC1, P7, CP5, CP3, C3, P5, C5, FC3, F3, F1, FCz, Fpz, C2, AF3, Fp1, FC2, FT7, T7, FC5, F2, Fz, TP7, O1, PO3, F5, F4, F7, AF4, CP2, FC4 Fz, AF3, AF4, FT8, Fp2, F4, F1, F2, O2, F8, FCz, FC4, P4, Cz, FC1, FC2, FC6, F6, AF8, T8, C2, P2, PO4, C1, CPz, F3, CP2, FC3, C3, C4, C6, TP8, POz, CP1, CP6, CP4, P6, P8, Pz, PO8, Oz, F5, Fpz, F7, PO3, PO7 CPz, CP2, Pz, P7, CP1, C1, P1, P2, POz, Cz, C2, FC4, PO4, P5, P4, Fz, FC2, CP6, FCz, CP4, C4, P3, O2, P8, F2, CP3, PO3, PO8, Oz, F4, O1, FC6, P6, FC1, F7, AF4, PO7, F3, F5, F1, F6, FT8, T8, C6, TP8, C3, FC3, TP7, CP5, Fp2, C5, AF3, FC5, T7, AF7, FT7, F8, AF8	CP5 at 592 ms (*F* = 25.11 *p* = 0.0096) CP6 at 204 ms (*F* = 36.22 *p* = 0.0498) C1 at 632 ms (*F* = 39.77 *p* = 0.0028)

**Table 4:** Summary of the main effects, interactions (*p* < 0.05) and follow-up comparisons (*p* < 0.0083) for the advice decision stage.

Test	Timing	Electrodes	Peak
Facial attractiveness main effect (P300) Facial attractiveness main effect (LPP) Facial attractiveness and feedback type interaction effect (N2) Acceptance: High facial attractiveness compared to low facial attractiveness Reject: High facial attractiveness compared to low facial attractiveness Facial attractiveness and feedback type interaction effect (LPP) Acceptance: High facial attractiveness compared to low facial attractiveness Reject: High facial attractiveness compared to low facial attractiveness	270–340 ms 500–800 ms 230–260 ms 500–800 ms	Cz, CPz, Pz, CP1, P1, C1, C2, CP2 Cz, CPz, Pz, CP1, P1, C1, C2, CP2 Fz, FCz, Cz, FC1, FC2, F1, F2, C1, C2 Cz, CPz, Pz, CP1, CP2, P2, C1, C2, P1	CP1 at 310 ms (*F* = 15.68 *p* = 0.0016) C1 at 630 ms (*F* = 13.49 *p* = 0.0034) F2 at 246 ms (*F* = 14.32 *p* = 0.0056) F2 at 246 ms (*F* = 11.41 *p* = 0.0070) N/A C1 at 632 ms (*F* = 39.77 *p* = 0.0022) C1 at 632 ms (*F* = 43.36 *p* = 0.0004) N/A

## Data Availability

The data presented in this study are available on request from the corresponding author due to participant privacy and ethical restrictions.
